# Development and Functional Characterization of Monoclonal Antibodies for Botulinum Neurotoxin Serotype A

**DOI:** 10.3390/foods14101743

**Published:** 2025-05-14

**Authors:** Jingmei Kang, Qingyu Lv, Wenhua Huang, Hua Jiang, Shan Gao, Qian Li, Decong Kong, Guofen Zhao, Peng Liu, Yongqiang Jiang

**Affiliations:** 1 Key Laboratory of Germplasm Innovation and Utilization of Triticeae Crop at Universities of Inner Mongolia Autonomous Region, College of Life Sciences, Inner Mongolia Agricultural University, Hohhot 010011, China; kangjingmei0211@163.com; 2 State Key Laboratory of Pathogen and Biosecurity, Academy of Military Medical Sciences, Beijing 100071, China; lvqingyu2004@126.com (Q.L.); huangwh1993@163.com (W.H.); jhua76@126.com (H.J.); gaoshan845@163.com (S.G.); liqian_bime@163.com (Q.L.); kongdecong-118@163.com (D.K.); ammsliupeng@163.com (P.L.)

**Keywords:** BoNT/A, TRFIA, rabbit mAb

## Abstract

Botulinum neurotoxin serotype A (BoNT/A), the most toxic of the seven serotypes produced by Clostridium botulinum, poses significant public health risks because of its involvement in foodborne outbreaks and potential use in bioterrorism. In this study, we developed high-affinity monoclonal antibodies for BoNT/A detection using single-cell fluorescence-activated cell sorting and nested PCR. The optimized antibody pair demonstrated exceptional sensitivity, detecting recombinant BoNT/A at concentrations as low as 0.02 ng/mL with a linear range of 0.02–10 ng/mL, while maintaining high specificity against BoNT/B, E, and F. Biolayer interferometry confirmed superior binding kinetics, and a time-resolved fluoroimmunoassay (TRFIA) demonstrated consistent performance in complex food matrices, including ham sausage and soybean paste. These rabbit-derived monoclonal antibodies enable ultrasensitive detection of BoNT/A across diverse food matrices, offering a powerful tool for food safety monitoring and biosecurity.

## 1. Introduction

*Clostridium botulinum*, a Gram-positive, spore-forming, and anaerobic bacterium, produces a 150 kDa inactive polypeptide precursor during replication. This precursor undergoes proteolytic cleavage to form botulinum neurotoxin (BoNT), a functionally active heterodimer composed of a 50 kDa light chain and a 100 kDa heavy chain, linked by a disulfide bond [1]. BoNT enzymatically targets proteins essential for the fusion of neurons and vesicles, inhibiting acetylcholine release and, thereby, acting as a pathogenic agent [2,3]. Recognized as one of the most lethal biological substances, BoNT has been classified as a Category A bioterrorism agent by the U.S. Centers for Disease Control and Prevention (CDC) [4,5]. The BoNT family includes seven well-characterized serotypes (A–G), over 40 subtypes, and a recently identified serotype H. Among these, serotype A exhibits the highest toxicity [6,7,8]. Toxicological studies in primates estimate human lethal doses (for a 70 kg body weight) at 0.70–0.90 μg (inhalation), 0.09–0.15 μg (intramuscular/intravenous), and 70 μg (oral ingestion) [9]. In addition to its potential use as a bioweapon, BoNT contamination in food matrices, such as salad dressing [10], processed meats [11], and fermented sauces [12], presents substantial public health risks. Therefore, developing rapid, sensitive, and reliable detection methods for BoNT is vital for both biodefense and food safety applications.

Advancements in detection technologies have led to the development of numerous methods for BoNT detection. While in vivo bioassays using animal models remain a gold standard, they are time-consuming and require specialized equipment and trained personnel. In contrast, alternative methods such as ELISA, PCR, cell-based assays, flow cytometry, mass spectrometry, and immunochromatographic assays significantly reduce detection times. Of these, immunochromatographic assays are particularly notable for their operational simplicity and potential for rapid, on-site detection. However, their effectiveness depends on the availability of high-sensitivity antibody pairs [13,14]. Therefore, developing and screening highly sensitive antibodies against BoNT is essential to improve detection capabilities.

In 1975, molecular biologists Köhler and Milstein revolutionized the field by isolating hybridoma cells that could secrete specific antibodies [15], marking the beginning of the monoclonal antibody era. Since then, scientific advancements have led to the development of innovative techniques for monoclonal antibody production, including hybridoma technology [16], phage display system [17], and single-B-cell technology [18]. These innovations have significantly enhanced the efficiency and precision of monoclonal antibody development. Among them, single-B-cell technology has gained widespread adoption because of its ability to reduce experimental timelines while maintaining high target specificity.

Rabbit monoclonal antibodies demonstrate superior binding affinity compared to their murine counterparts. In recent years, the approach of preparing rabbit monoclonal antibodies based on single-B-cell sorting technology has been steadily evolving. Currently, the prevalent method involves culturing the sorted cells for 7 days prior to amplifying the antibody variable region. This approach not only results in a long experimental cycle but also imposes stringent requirements on both the sorting environment and the culture conditions for rabbit single cells. The nested PCR method has been extensively applied to amplify the variable regions of single B cells in mice and humans. However, its application in rabbits has not been reported thus far.

In this study, we designed a set of primers for nested PCR specifically tailored for the amplification of the rabbit antibody variable region. This design allows for the rapid acquisition of the variable region sequence within 48 h after cell sorting. Our approach integrates fluorescence-activated cell sorting (FACS) for isolating antigen-specific single B cells with nested PCR amplification of paired variable regions from both heavy and light chains. Through systematic screening with time-resolved fluoroimmunoassay (TRFIA) using europium-labeled microspheres, we identified optimal rabbit monoclonal antibody pairs to detect BoNT/A in this study. This methodological advancement has enabled the creation of a rapid detection platform for BoNT/A, offering both high sensitivity and rapid detection capabilities for BoNT/A identification.

## 2. Materials and Methods

### 2.1. Expression and Purification of BoNT Recombinant Proteins

The nucleotide sequence encoding the BoNT/A Hc fragment was cloned into the pET-32a (+) vector between *NdeI* and *XhoI* restriction sites, generating the recombinant plasmid. This construct was then transformed into *E. coli* BL21(DE3) (TransGen Biotech, Beijing, China). Positive clones were selected and cultured overnight in 5 mL LB broth (Becton, Franklin Lakes, NJ, USA) supplemented with 50 µg/mL kanamycin under shaking conditions. The culture was subsequently scaled up to 500 mL of LB medium with 50 µg/mL kanamycin at 37 °C. When the optical density at 600 nm (OD_600_) reached 0.6–0.8, protein expression was induced by adding 1 mM isopropyl β-D-1-thiogalactopyranoside (IPTG), followed by incubation at 28 °C for 4 h. Cells were harvested by centrifugation at 8000 rpm for 10 min, and the pellet was resuspended in 100 mL lysis buffer (20 mM phosphate buffer, 500 mM NaCl, 20 mM imidazole, and pH 7.4). After sonication, the mixture was centrifuged at 8000 rpm for 10 min, and the supernatant was collected for protein purification.

The recombinant BoNT/A antigen was purified using an ÄKTA purification system (Cytiva, Wilmington, DE, USA) with a 5 mL HisTrap HP column (Cytiva, Wilmington, DE, USA). Following purification, the protein was dialyzed overnight against 20 mM phosphate buffer and 150 mM NaCl at pH 7.4 (PBS) at 4 °C. The protein concentration was then quantified using a NanoDrop 2000 spectrophotometer (Thermo Fisher Scientific, Waltham, MA, USA). Using this protocol, recombinant antigens of BoNT/B, BoNT/E, and BoNT/F were also expressed.

### 2.2. Animal Immunization and Single-B-Cell Isolation via Flow Cytometry

New Zealand White rabbits (Beijing Jinmuyang Laboratory Animal Breeding, Beijing, China) underwent four immunizations according to the schedule in Table 1. One week after the final immunization, blood was drawn from the marginal ear vein for serum collection. ELISA was used to determine serum antibody titers, with the ELISA plates coated with 1 μg/mL recombinant BoNT/A antigen and blocked with 3% (*w*/*v*) BSA. Serial 10-fold serum dilutions were tested. When the titers reached 10^7^–10^8^, an additional 20 mL blood sample was collected from the marginal ear vein.

Peripheral blood mononuclear cells (PBMCs) were isolated using Lympholyte^®^-Mamma density gradient medium (Cedarlane, Hornby, ON, Canada). Briefly, 15 mL of Lympholyte^®^-Mamma was added to centrifuge tubes, followed by layering with a 1:1 mixture of blood and PBS. After centrifugation at 1000 rpm for 20 min, the PBMC layer was collected and washed with 40 mL HBSS buffer (1% FBS, 1 mM EDTA, and 25 mM HEPES) at 1000 rpm for 10 min. Red blood cells were lysed using 10× RBC Lysis Buffer (BioLegend, San Diego, CA, USA), followed by multiple washes. The final cell pellet was resuspended in 1 mL HBSS buffer for counting.

For cell staining, 1 × 10^7^ cells were incubated with 1 μL each of APC- and PE-conjugated BoNT/A antigen (Lightning-Link^®^, Abcam, Cambridge, MA, USA), 0.5 μL each of anti-rabbit CD4/CD8/Pan-T lymphocyte antibodies (FITC) (Bio-Rad, Hercules, CA, USA), 1 μL of anti-rabbit IgM (PE/Cy7) (Lightning-Link^®^, Abcam, Cambridge, MA, USA), and 1 μL of anti-rabbit IgG (BV421) (Becton, Franklin Lakes, NJ, USA). After a 30 min incubation at room temperature, cells were washed, analyzed, and sorted using an LSRFortessa flow cytometer (Becton, Franklin Lakes, NJ, USA). To distinguish live from dead cells, 1 μL of 7-AAD viability dye (Thermo Fisher Scientific, Waltham, MA, USA) was added before sorting. Target B cells were identified based on the following fluorescence profile: 7-AAD(PerCP-Cy5)-/CD4:FITC-/CD8:FITC-/Pan-T:FITC-/IgM:PE-Cy7-/IgG:BV421-/antigen:APC+/antigen:PE+. Single target cells were sorted into 96-well plates preloaded with single-cell lysis buffer (Tianjingsha Gene, Beijing, China), with one cell per well. Sorted plates were immediately snap-frozen on dry ice and stored at −80 °C for subsequent cDNA synthesis.

### 2.3. Amplification and Expression of Rabbit Monoclonal Antibodies Against BoNT/A

For cDNA synthesis, each well containing single cells was supplemented with 5 μL of 4× All-in-One RT Master Mix (ALLMEEK, Beijing, China) and 10 µL nuclease-free water. Reverse transcription was conducted at 50 °C for 15 min, followed by enzyme inactivation at 90 °C for 1 min. The variable regions of the heavy and light chains were amplified using nested PCR with specific primers (Table 2). The primary PCR reaction included an initial denaturation at 94 °C for 3 min, followed by 30 cycles of denaturation at 94 °C for 25 s, annealing at 55 °C for 25 s, and extension at 72 °C for 25 s, with a final extension at 72 °C for 5 min. For the secondary (nested) PCR, 2 μL of unpurified primary PCR product was used as a template with the same cycling parameters, except for an increased cycle number (35 cycles instead of 30).

Secondary PCR products were analyzed via 1.5% (*w*/*v*) agarose gel electrophoresis and visualized under UV illumination. The amplified PCR products were then subjected to Sanger sequencing (Tianyihuiyuan, Beijing, China). Successfully amplified antibody sequences underwent sequential analysis, from which sixteen paired sequences were selected for full-length antibody expression plasmid construction. The variable heavy (VH)- and light (VL)-chain sequences were cloned into eukaryotic expression vectors containing the rabbit IgG heavy-chain constant region and kappa light-chain constant region. The recombinant plasmids were transformed into Trans-T1 competent cells (TransGen Biotech, Beijing, China) and plated on ampicillin-containing agar. Positive clones were selected for liquid culture, and plasmid DNA was extracted using an endotoxin-free plasmid extraction kit (TransGen Biotech, Beijing, China).

For antibody production, expression plasmids were transfected into Expi293F cells (Thermo Fisher Scientific, Waltham, MA, USA) using Expifectamine™ 293 transfection reagent. After one week of culture, the supernatant was collected by centrifugation at 8000 rpm for 10 min. Antibody purification was performed using an ÄKTA pure chromatography system (Cytiva, Wilmington, DE, USA). Protein A affinity chromatography was carried out with a binding buffer consisting of 20 mM phosphate buffer (PB) and 0.15 M NaCl at pH 7.4. After loading and washing the sample, the bound antibodies were eluted with 20 mM sodium acetate buffer (pH 3.5) and immediately neutralized with 1 M Tris-HCl (pH 9.0). The purified antibodies were dialyzed overnight against PBS at 4 °C, and the protein concentration was quantified using a NanoDrop 2000 spectrophotometer (Thermo Fisher Scientific, Waltham, MA, USA). Antibody purity and integrity were assessed by SDS-PAGE under denaturing conditions.

### 2.4. Biolayer Interferometry Analysis of Monoclonal Antibody Binding Kinetics

The binding activity and kinetics of selected monoclonal antibodies to recombinant BoNT/A antigen were further evaluated using a Biolayer Interferometry (BLI) assay on a Gator Prime system (Gator Bio, Suzhou, Jiangsu, China), following the manufacturer’s instructions. Data analysis was performed using Gator software (Gator One 2.17.7.0416). Monoclonal antibodies with rabbit IgG constant regions were captured on Protein A biosensors and were then immersed in the following solutions: (1) analyte wells containing 100 nM recombinant BoNT/A antigen in binding buffer (PBS containing 0.02% Tween 20 and 0.2% BSA) to measure the association rate (on-rate, kon) and, subsequently, (2) wells containing binding buffer to assess the dissociation rate (off-rate, koff). The binding affinity constants (dissociation constant, KD) were determined as koff/kon.

### 2.5. Preparation of Time-Resolved Fluorescence Microsphere-Labeled Antibodies

A total of 500 µg Eu (III) chelate microparticles were diluted with 1 mL 50 mM MES buffer (pH 6.0). A total of 35 µg 1-ethyl-3-(3-dimethylaminopropyl) carbodiimide (EDC) and 350 µg N-hydroxysuccinimide (NHS) were added to the microparticle suspension, which was then incubated at room temperature for 20 min. After incubation, the suspension was centrifuged at 14,500 rpm for 15 min, and the supernatant was discarded. The microparticles were washed twice with 1 mL of 50 mM MES buffer (pH 6.0) and re-suspended in 1 mL of the same buffer. The suspension was homogenized using a sonicator until it became clear and free of visible particles. Next, 50 µg of monoclonal antibodies (mAbs) to recombinant BoNT/A antigen were added to the microparticle suspension. The antibody–microparticle mixture was incubated at room temperature for 2 h. To block unbound spots on the microparticles, 25 µL of 20% (*w*/*v*) BSA in 50 mM Tris-HCl was added to the suspension The blocking reaction was incubated at room temperature for 1 h. The microparticle-conjugated antibodies were then washed twice with 50 mM Tris-HCl (pH 8.0) and re-suspended in 500 µL of 50 mM Tris-HCl (pH 8.0). The final suspension was stored at 4 °C for future experiments.

### 2.6. Preparation of Time-Resolved Fluoroimmunoassay Strips

The TRFIA system consists of the following four components: PVC base, nitrocellulose (NC) membrane, sample pad, and absorbent pad. Staphylococcal protein A (SPA) and BoNT/A antibody were each diluted to 1 mg/mL with PBS. The quality control line (C line) was coated with 1 mg/mL of SPA, and the detection line (T line) was coated with 1 mg/mL of BoNT/A antibody. The time-resolved fluorescent microsphere-labeled antibody and the time-resolved fluorescent microsphere preservation solution were mixed at a 1:4 ratio, and the mixture was evenly applied to the sample pads. The pads were then dried in an oven for 30 min. After drying, the assembled test strips were cut into 3.6 mm wide sections.

### 2.7. Time-Resolved Fluoroimmunoassay Test Procedure

The test procedure was as follows: 100 μL of test sample was applied to the sample pad of the strip. After incubation for 15 min at room temperature, the strip was inserted into a fluorescent immunoassay analyzer (Hangzhou Autokun Technology Co., Ltd., Hangzhou, Zhejiang, China). The analyzer measured the fluorescence intensities of the test (T) and control (C) lines and calculated the T/C ratio (the ratio of the test line to the control line fluorescence).

### 2.8. Evaluation of Time-Resolved Fluoroimmunoassay

The sensitivity and linear range of TRFIA were evaluated by performing a two-fold serial dilution of the 10 ng/mL recombinant BoNT/A antigen. The serial concentrations of recombinant BoNT/A antigen were plotted on the *x*-axis, while the corresponding T/C ratios were plotted on the *y*-axis. Subsequently, a linear regression analysis was performed to generate the standard curve, which was used to determine the sensitivity and linear range. The cut-off value was calculated as the mean T/C ratio of negative controls (0 ng/mL antigen in dilution buffer) plus three standard deviations (mean + 3SD). The limit of detection (LOD) was defined as the lowest antigen concentration with a mean T/C ratio significantly exceeding the cut-off value. To assess the specificity, the cross-reactivity was evaluated using recombinant BoNT/A, BoNT/B, BoNT/E, and BoNT/F antigens (10, 50, and 100 ng/mL). To evaluate the reproducibility, recombinant BoNT/A antigen was tested at 0.1, 1, and 10 ng/mL (*n* = 12 replicates per concentration).

### 2.9. Preparation of Simulated Samples

For the matrix effect evaluation, commercially available ham sausage and soybean paste were purchased from local supermarkets. A total of 0.4 g of each sample was weighed into 1.5 mL microcentrifuge tubes, followed by the addition of 1 mL of dilution buffer (50 mM phosphate buffer containing 2% BSA and 0.1% Tween-20, pH 7.4). The samples were homogenized using a tissue homogenizer at 70 Hz for 180 s. The homogenates were then centrifuged at 8000 rpm for 10 min at 4 °C, and the supernatants were collected as 40% (*w*/*v*) stock solutions. Serial dilutions were prepared with dilution buffer to obtain 10% and 20% (*w*/*v*) working solutions.

For spiked sample preparation, known concentrations of recombinant BoNT/A antigen and BoNT/A crude toxin were added to the 10% and 20% (*w*/*v*) matrix solutions, creating a series of simulated samples with varying toxin concentrations for subsequent analytical validation. BoNT/A crude toxin was prepared as described in [19]. Given the high toxicity of native BoNT/A, appropriate safety measures were implemented. All BoNT/A crude toxin handling occurred within a class 2 biosafety cabinet equipped with HEPA filters. All used materials (test strips, centrifuge tubes, pipette tips, etc.) were sterilized by autoclaving.

### 2.10. Statistical Analysis

Data are expressed as the mean ± standard deviations (SD). The Student’s *t*-test was used to compare two groups. A *p*-value of < 0.05 was considered statistically significant.

## 3. Results

### 3.1. Design of Nested PCR Primers for Amplifying the Variable Regions of Rabbit Antibodies

Rabbit antibodies can be classified into five categories (IgG, IgM, IgA, IgE, and IgD) based on their heavy chain differences. Among these, IgG is the most abundant isotype in rabbits, and no IgG subtypes (e.g., IgG1 and IgG2a) have been identified in this species. Rabbit antibody light chains are classified into the following three types: κ1, κ2, and λ. Approximately 90–95% of rabbit light chains are κ1, with κ2 and λ collectively accounting for 5–10%. Therefore, nested PCR primers were designed to amplify rabbit IgG heavy chains and κ1 light chains. All sequences for IGHV, IGHC (IgG constant region), IGKV, and IGKC (κ constant region) were curated from the IMGT database and aligned to identify conserved regions. Upstream primers targeted the ‘L-PART1’ region of IGHV and IGKV, while downstream primers were designed within IGHC and IGKC. The primers’ details are listed in Table 2, and their binding sites are illustrated in Figures S1 and S2.

### 3.2. Expression of Recombinant BoNT/A Protein, Single-B-Cell Isolation, and Antibody Variable Region Amplification

**Figure 1:**
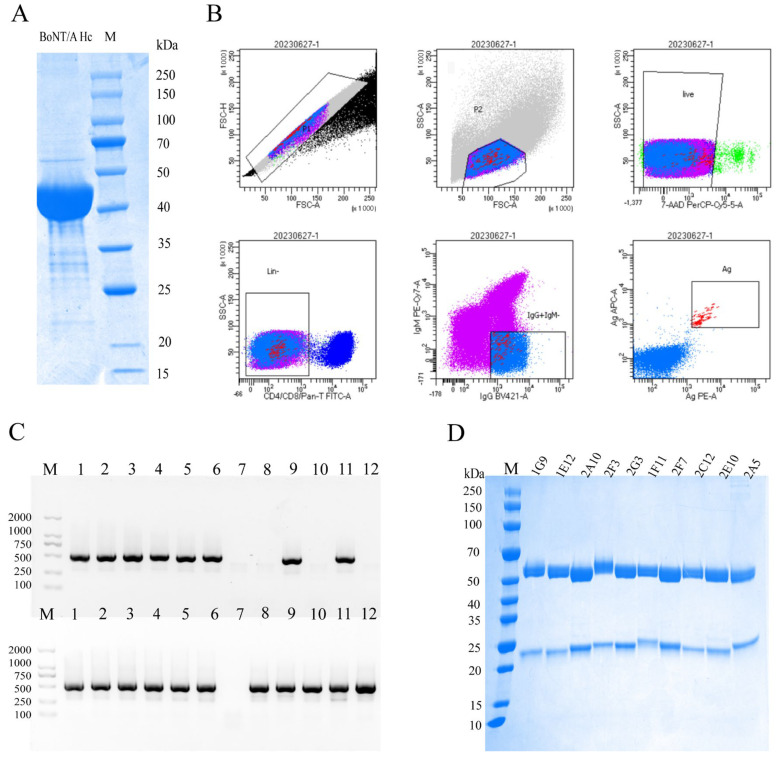
Integrated bioprocessing workflow for the development of the BoNT/A-specific monoclonal antibody, from antigen purification to recombinant antibody production. (**A**) SDS-PAGE analysis of the recombinant BoNT/A antigen (BoNT/A Hc). M: Protein marker (kDa). (**B**) Isolation of antigen-specific B lymphocytes via fluorescence-activated cell sorting. (**C**) Molecular characterization of antibody variable regions by nested PCR. (**Upper**) Heavy-chain variable region amplicon. (**Lower**) Light-chain variable region amplicon. M: DNA ladder (100–2000 bp). (**D**) Quality assessment of purified monoclonal antibodies. The upper band represents the heavy chain (~50 kDa), while the lower band corresponds to the light chain (~25 kDa). M: Protein marker (kDa).

The recombinant BoNT/A antigen (45 kDa) was expressed in *Escherichia coli*, with its molecular weight confirmed by SDS-PAGE (Figure 1A). New Zealand White rabbits were immunized four times with purified recombinant BoNT/A antigen (two-week intervals), and peripheral blood was drawn from the ear marginal vein for single-B-cell isolation (Figure 1B). For FACS, lymphocytes were initially gated, excluding doublets and debris. Viable cells were selected, and T cells were depleted. The population was further gated for IgM:PE-Cy7-/IgG:BV421+-positive cells, and BoNT/A-binding B lymphocytes were, ultimately, selected as target cells. Single cells were sorted into 96-well plates containing lysis buffer.

Following cDNA synthesis, variable regions of the heavy and light chains were amplified by nested PCR. Agarose gel electrophoresis of the PCR products showed amplification bands of approximately 500 bp (Figure 1C). The secondary PCR products were then subjected to Sanger sequencing, and paired heavy- and light-chain sequences were successfully obtained from approximately 70% of the cells.

### 3.3. Purification and Screening of High-Affinity Rabbit Monoclonal Antibodies Against BoNT/A

Sixteen antibodies were randomly selected for plasmid construction, recombinant expression, and purification. Their binding affinities were quantified by biolayer interferometry (BLI). Six clones showing suboptimal characteristics were excluded, while ten high-affinity clones (1G9, 1E12, 2A10, 2F3, 1F11, 2F7, 2C12, 2E10, 2A5, and 2G3) were selected. All ten exhibited KD values < 10^−9^ M (Table 3). Notably, 2A10 demonstrated particularly strong binding to the probe, with minimal dissociation observed over the experimental timeframe, preventing accurate koff determination. SDS-PAGE under reducing conditions (5% β-mercaptoethanol) confirmed expected IgG structure, with heavy (~50 kDa) and light chains (~25 kDa) (Figure 1D). These high-affinity antibodies show considerable potential for diagnostic and therapeutic applications targeting BoNT/A.

### 3.4. Screening of Paired Antibodies Using TRFIA

**Figure 2:**
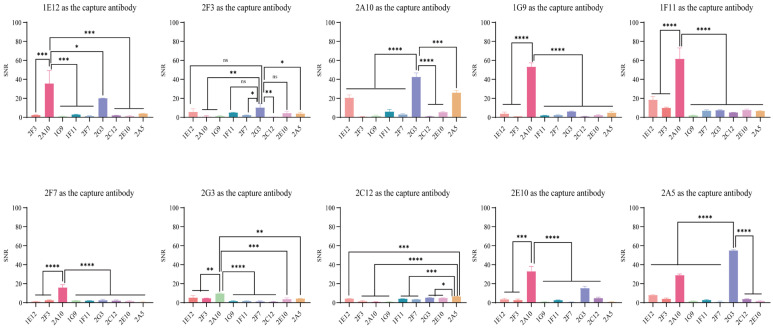
Screening of paired antibodies using TRFIA. Signal-to-noise ratio (SNR) was calculated as (mean T/C ratio of 1 ng/mL BoNT/A samples)/(mean T/C ratio of negative controls). Significance levels: ns (not significant), * *p* < 0.05, ** *p* ≤ 0.01, *** *p* ≤ 0.001, **** *p* ≤ 0.0001.

All possible pairwise combinations of the ten purified antibodies were tested using a time-resolved fluoroimmunoassay. One antibody was immobilized on nitrocellulose test lines as the capture antibody, while the other antibody was conjugated to time-resolved fluorescent microspheres and applied to the sample pad as the detection antibody. For the assay, 1 ng/mL recombinant BoNT/A antigen and dilution buffer were added to the TRFIA strips, which were then incubated for 15 min. Subsequently, the T/C ratios were determined, and the signal-to-noise ratio (SNR) was calculated. Figure 2 presents the pairwise screening results. The highest SNR of 61.60 was observed when 1F11 served as the capture antibody and 2A10 as the detection antibody. The second- and third-highest SNR values were 55.00 (2A5 as the capture antibody paired with 2G3 as the detection antibody) and 53.36 (1G9 as the capture antibody paired with 2A10 as the detection antibody), respectively. Given its superior SNR, the 1F11 (capture) and 2A10 (detection) antibody pair was selected for further performance evaluation.

### 3.5. Sensitivity, Specificity, and Reproducibility of TRFIA

**Figure 3:**
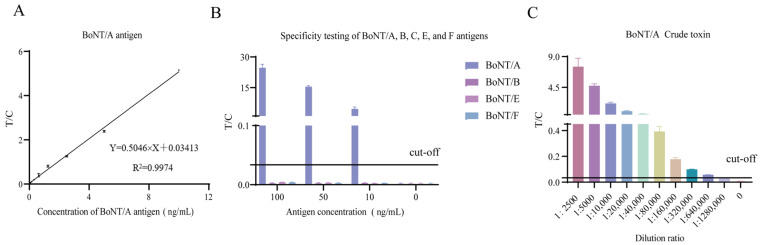
Sensitivity and specificity evaluations of selected antibody pairs: (**A**) sensitivity assessment for recombinant BoNT/A antigen detection; (**B**) cross-reactivity testing against BoNT serotypes A, B, C, E, and F; (**C**) sensitivity assessment for crude BoNT/A toxin detection. The cut-off value was defined as the mean T/C ratio of negative controls plus 3 standard deviations (mean + 3SD).

The antibody pair (1F11 and 2A10) with the highest SNR from the previous screening was used to evaluate the detection sensitivity. A two-fold serial dilution of 10 ng/mL recombinant BoNT/A antigen in buffer was prepared for this purpose. The standard curve was generated by performing linear regression analysis of the concentration values versus the T/C ratios. The results reveal a limit of detection (LOD) of 0.02 ng/mL for recombinant BoNT/A antigen, with a linear range from 0.02 ng/mL to 10 ng/mL (Figure 3A). To assess specificity, recombinant BoNT/A, BoNT/B, BoNT/E, and BoNT/F antigens were diluted to 100 ng/mL, 50 ng/mL, and 10 ng/mL in buffer, respectively. The time-resolved fluoroimmunoassay showed no cross-reactivity with BoNT/B, BoNT/E, or BoNT/F, confirming high specificity for BoNT/A (Figure 3B). For native toxin detection, BoNT/A crude toxin was initially diluted 1:2500 and then a two-fold serial dilution was performed. The results indicate an LOD of 1:640,000 for BoNT/A crude toxin (Figure 3C), demonstrating that the selected antibody pair effectively detects and identifies the native toxin. To further assess reproducibility, recombinant BoNT/A antigen was tested at concentrations of 0.1, 1, and 10 ng/mL, diluted in buffer, across 12 replicates. The coefficient of variation (CV) for all three concentrations was less than 10%, indicating excellent reproducibility and homogeneity of the assay (Table 4).

### 3.6. Detection of Simulated Samples

**Figure 4:**
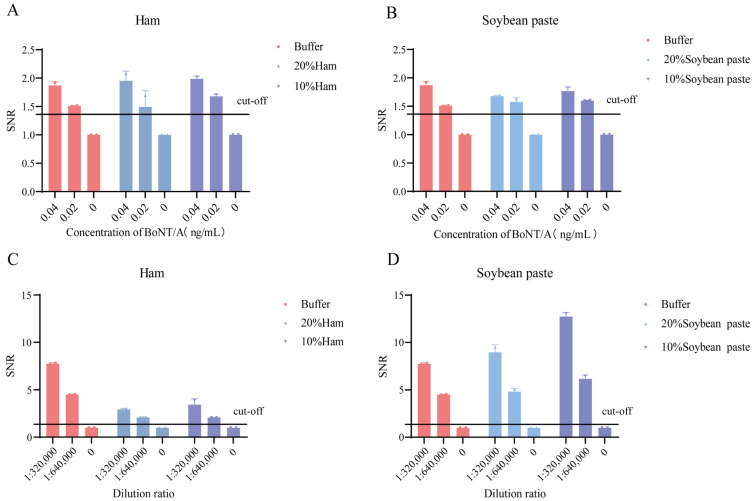
Detection of BoNT/A in simulated food matrices. Detection of (**A**) recombinant BoNT/A in ham, (**B**) recombinant BoNT/A in bean paste, (**C**) crude BoNT/A toxin in ham, and (**D**) crude BoNT/A toxin in bean paste. Signal-to-noise ratio (SNR) was calculated as follows: (mean T/C ratio of spiked samples)/(mean T/C ratio of unspiked matrix controls). The cut-off value was calculated as the mean T/C ratio of unspiked matrix controls plus 3 standard deviations, divided by the mean T/C ratio of unspiked matrix controls.

The impact of different sample matrices on the detection performance of the 1F11 and 2A10 antibody pair was assessed by spiking recombinant BoNT/A antigen and BoNT/A crude toxin into processed ham sausage and soybean paste extracts diluted in buffer. Using TRFIA strips fabricated with the selected antibody pair, the detection assays were conducted. The results (Figure 4) show that the LOD for recombinant BoNT/A antigen remained at 0.02 ng/mL in both 20% and 10% diluted ham sausage and soybean paste matrices, consistent with the LOD in buffer. Similarly, the LOD for BoNT/A crude toxin in these matrices was 1:640,000, identical to that observed in buffer. These findings confirm that the high-affinity antibody pair maintains its performance in complex matrices, demonstrating excellent matrix tolerance and suitability for detecting BoNT/A in diverse sample types.

## 4. Discussion

BoNT is widely recognized for its potent toxicity and potential as a bioweapon, but it also holds significant therapeutic value. In medicine, BoNT is commonly used to treat a variety of clinical conditions, including neurological disorders [20], urological diseases [21], and gastrointestinal disorders [22,23]. Its use in cosmetic treatments, such as facial contouring [24] and wrinkle reduction [25], is also well-established. However, due to its extreme toxicity—greater than that of agents like Novichok, Sarin, and Ricin [26]—improper dosage can lead to severe health risks, including coma, shock, and death. Thus, while BoNT/A can have a significant positive impact on human health, whether used as a bioweapon or for therapeutic purposes, it carries substantial risks. This underscores the urgent need for rapid, highly sensitive, and accurate detection methods for BoNT.

Several methods for detecting BoNT have been developed. The enzyme-linked immunosorbent assay (ELISA), with a detection limit of 5 pg to 2 ng/mL [13], is commonly used. While ELISA is relatively sensitive, its complex procedures, such as plate washing, make it unsuitable for on-site detection. The in vivo mouse bioassay, which offers a detection limit of 1–10 pg/mL, provides high sensitivity but is time-consuming (up to 4 days) and requires animal testing [27], making it impractical for field use. The lateral flow assay, known for its simplicity, has a detection limit of 10–50 ng/mL [27,28]. This method relies on antigen–antibody interactions to form visible bands on test strips. However, its use of gold nanoparticles as labels results in limited stability, low sensitivity, and an inability to provide quantitative results [29]. Mass spectrometry offers significantly higher sensitivity (0.1–1 pg/mL) [27], outperforming ELISA and lateral flow assays by 10- to 100-fold while maintaining strong specificity. However, its reliance on expensive equipment and complex protocols limits practical application. Similarly, microfluidic-based sandwich immunoassays achieve a detection limit of 30 pg/mL with minimal sample consumption (5 μL of serum) [30]. Despite its sensitivity, the assays require intricate microfluidic channel designs, which involve precise control over dimensions, curvature, and surface properties, increasing manufacturing complexity and cost [31].

In this study, we utilized TRFIA with lanthanide-doped fluorescent microspheres as labels to enhance sensitivity and stability [29,32]. Our results demonstrate that TRFIA provides rapid detection within 15 min and achieves a detection limit of 0.02 ng/mL for BoNT/A. Moreover, it exhibits strong specificity, as no cross-reactivity was observed with recombinant BoNT/B, BoNT/E, and BoNT/F antigens. This method combines high sensitivity, strong specificity, and rapid turnaround. Additionally, its portable instrumentation makes it well-suited for on-site BoNT/A detection.

In addition to detection method optimization, assay performance critically depends on antibodies with high affinity and specificity. Compared to murine monoclonal antibodies (mAbs), rabbit mAbs exhibit superior characteristics, as follows: (1) higher affinity (KD typically 10^−11^–10^−13^ M), (2) broader epitope recognition, and (3) lower background signals. These properties eliminate the need for in vitro affinity maturation and enable highly sensitive detection assays. Recent advances in single-B-cell technology have revolutionized antibody discovery. Our platform integrates FACS with nested PCR to obtain paired heavy-/light-chain sequences within 48 h post-sorting—a significant improvement over traditional hybridoma methods requiring months. This approach provides a robust pipeline for rapid rabbit mAb development, with broad applications in diagnostics and therapeutics.

Huang et al. [33] generated antibodies against ciprofloxacin by fusing rabbit spleen cells with myeloma cells using hybridoma technology, achieving an LOD of 0.095 ng/mL via indirect competitive ELISA. Similarly, Liang et al. [34] developed rabbit monoclonal antibodies against T-2 toxin using single-B-cell technology and applied an indirect competitive ELISA for detection in milk samples, achieving an LOD of 0.32 μg/kg. Inspired by these studies, we used single-B-cell technology to generate and screen rabbit monoclonal antibodies against BoNT/A. The selected BoNT/A antibodies exhibited high affinity, excellent specificity, and minimal interference from matrices. In addition to the optimal 1F11-2A10 antibody pair, two alternative pairs (2A5-2G3 and 1G9-2A10) also showed high signal-to-noise ratios, demonstrating their potential for BoNT/A detection and identification.

Compared to traditional hybridoma techniques, single-B-cell technology significantly reduces antibody production time and improves preparation efficiency, providing high-quality antibodies for immunoassays. However, this approach presents certain technical challenges, including flow cytometry-based single-B-cell sorting and nested PCR amplification of variable regions from heavy and light chains, which require specialized equipment and incur higher costs. The limitations of TRFIA should also be considered. While TRFIA offers advantages such as high quantum yield, low background fluorescence, and superior signal-to-noise ratio—characteristics shared with most immunochromatographic assays—it remains dependent on nitrocellulose (NC) membrane chromatography. For complex or large-particle matrices, this may cause significant background interference or membrane clogging, potentially leading to inaccurate results. Therefore, appropriate sample pretreatment is essential to minimize these effects.

## 5. Conclusions

In summary, this study produced 10 high-affinity, high-expression monoclonal antibodies against BoNT/A. We used a selected antibody pair to establish a rapid detection method based on time-resolved fluorescent microspheres, achieving fast and highly sensitive detection of BoNT/A. This method offers a novel approach for on-site rapid detection of BoNT/A.

## Figures and Tables

**Table 1:** Immunization programs.

Immunizing Time (Day)	Immunizing Dose (mg)	Immunizing Mode	Immunoadjuvant
1	0.25	Subcutaneous immunity	Complete Freund’s adjuvant
14	0.5	Muscle immunity	Incomplete Freund’s adjuvant
28	1	Muscle immunity	Incomplete Freund’s adjuvant
42	1.5	Muscle immunity	Incomplete Freund’s adjuvant

**Table 2:** Primer sequences used for variable region amplification of heavy and light immunoglobulin chains.

Primer	Nucleotide Sequence (5′→3′)	
VL-F1	ATGGACACSAGGGCCCCCACTC (S = C or G)	First PCR
VL-R1	GTRCTGCTGAGGTTGTAGGTA (R = A or G)	
VH-F1	GACTGGGCTGCGCTGGCTTCTCCT	
VH-R1	CATTGGTGAGGGTGCCCGAGT	
VL-F2	CTGCTGGGGCTCCTGCT	Second PCR
VL-R2	ATCCACCTYCCAGGTGACGG (Y = C or T)	
VH-F2	GGTCGCTGTGCTCAAAGGT	
VH-R2	ARGTCACGGTCACTGGCTC (R = A or G)	

**Table 3:** Binding kinetic parameters.

Monoclonal Antibodies	Koff (1/s)	Kon (1/Ms)	KD (M)
2A10	Undetectable	5.25 × 10^5^	<1 × 10^−12^
1G9	4.69 × 10^−6^	6.64 × 10^5^	7.07 × 10^−12^
2F7	2.40 × 10^−5^	3.80 × 10^5^	6.32 × 10^−11^
1E12	2.97 × 10^−5^	5.32 × 10^5^	5.58 × 10^−11^
1F11	4.62 × 10^−4^	6.37 × 10^5^	7.26 × 10^−10^
2F3	3.13 × 10^−4^	4.50 × 10^5^	6.96 × 10^−10^
2C12	4.63 × 10^−5^	4.14 × 10^5^	1.12 × 10^−10^
2A5	2.99 × 10^−3^	3.92 × 10^5^	7.63 × 10^−9^
2E10	4.89 × 10^−4^	1.52 × 10^5^	3.21 × 10^−9^
2G3	2.84 × 10^−4^	1.86 × 10^5^	1.53 × 10^−9^

**Table 4:** Reproducibility of TRFIA.

Concentration (ng/mL)	Mean ± SD (*n* = 12)	Coefficient of Variation (CV%)
10	4.57 ± 0.41	9.02
1	0.51 ± 0.05	9.86
0.1	0.05 ± 0.00	7.35

## Data Availability

The original contributions presented in this study are included in the article/Supplementary Material. Further inquiries can be directed to the corresponding authors.

## Supplementary Materials

The following supporting information can be downloaded at: https://www.mdpi.com/article/10.3390/foods14101743/s1, Figure S1: The positions of nested PCR primers used for heavy-chain amplification; Figure S2: The positions of nested PCR primers used for light-chain amplification. Figure S3: Screening of paired antibodies using TRFIA.
