# Supplementary material for: Development and Functional Characterization of Monoclonal Antibodies for Botulinum Neurotoxin Serotype A
Source: Foods. 2025 May 14;14(10):1743. doi: 10.3390/foods14101743 (PMC12111189; doi:10.3390/foods14101743)
Supplement: Supplementary file 1 [file foods-14-01743-s001.zip › foods-3593551-supplementary.pdf]

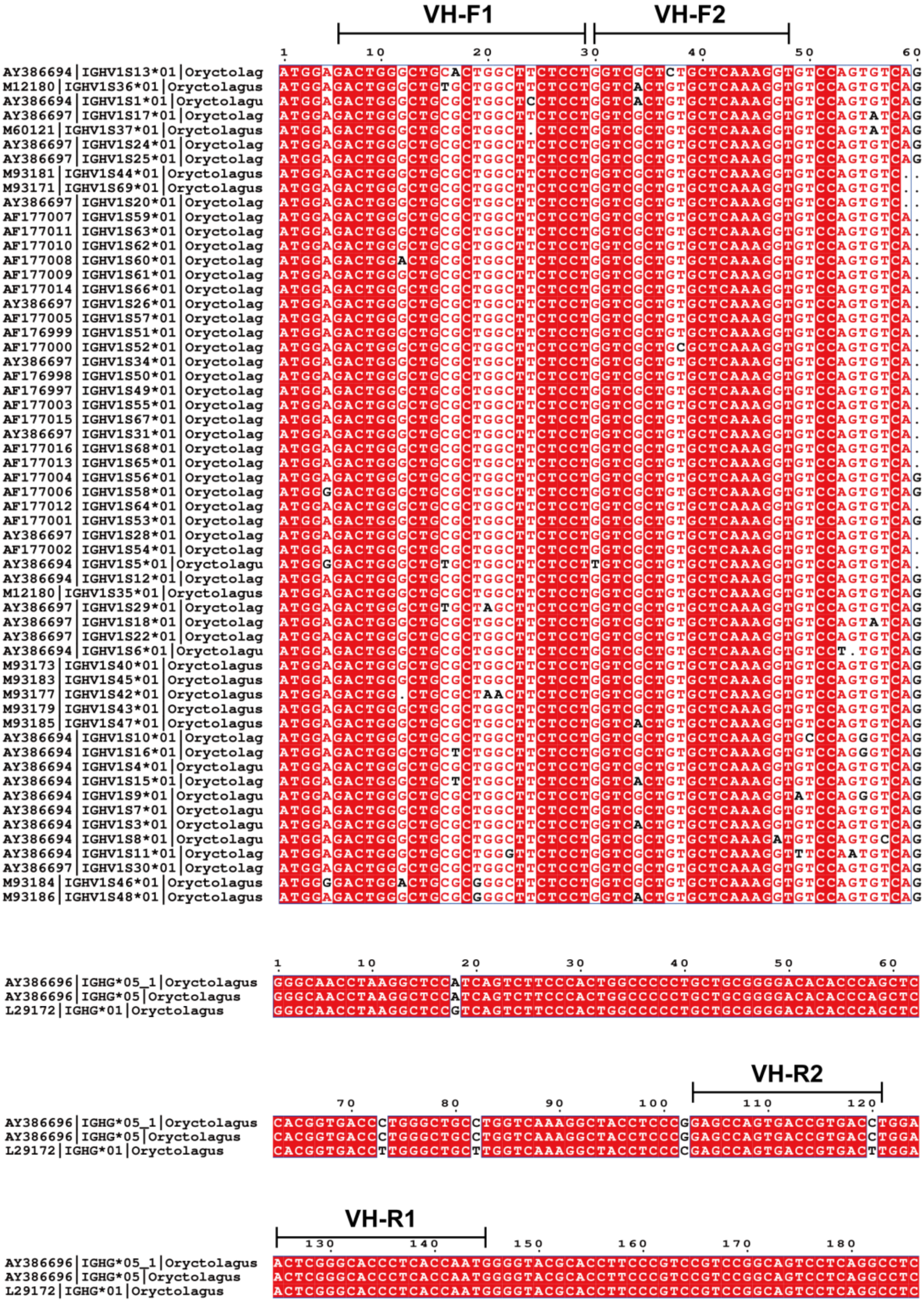

Figure S1 The positions of nested PCR primers used for heavy chain amplification.

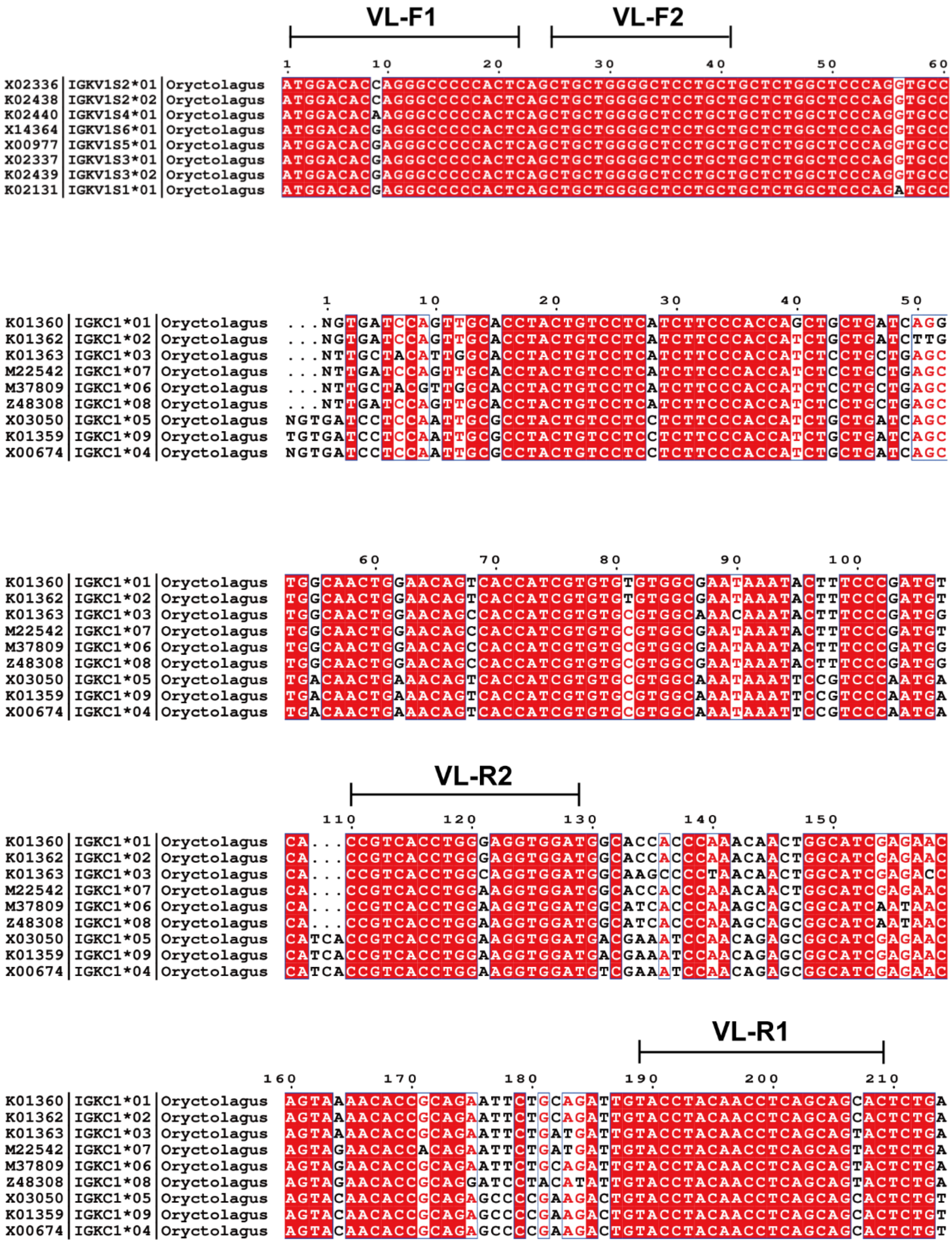

Figure S2 The positions of nested PCR primers used for light chain amplification.

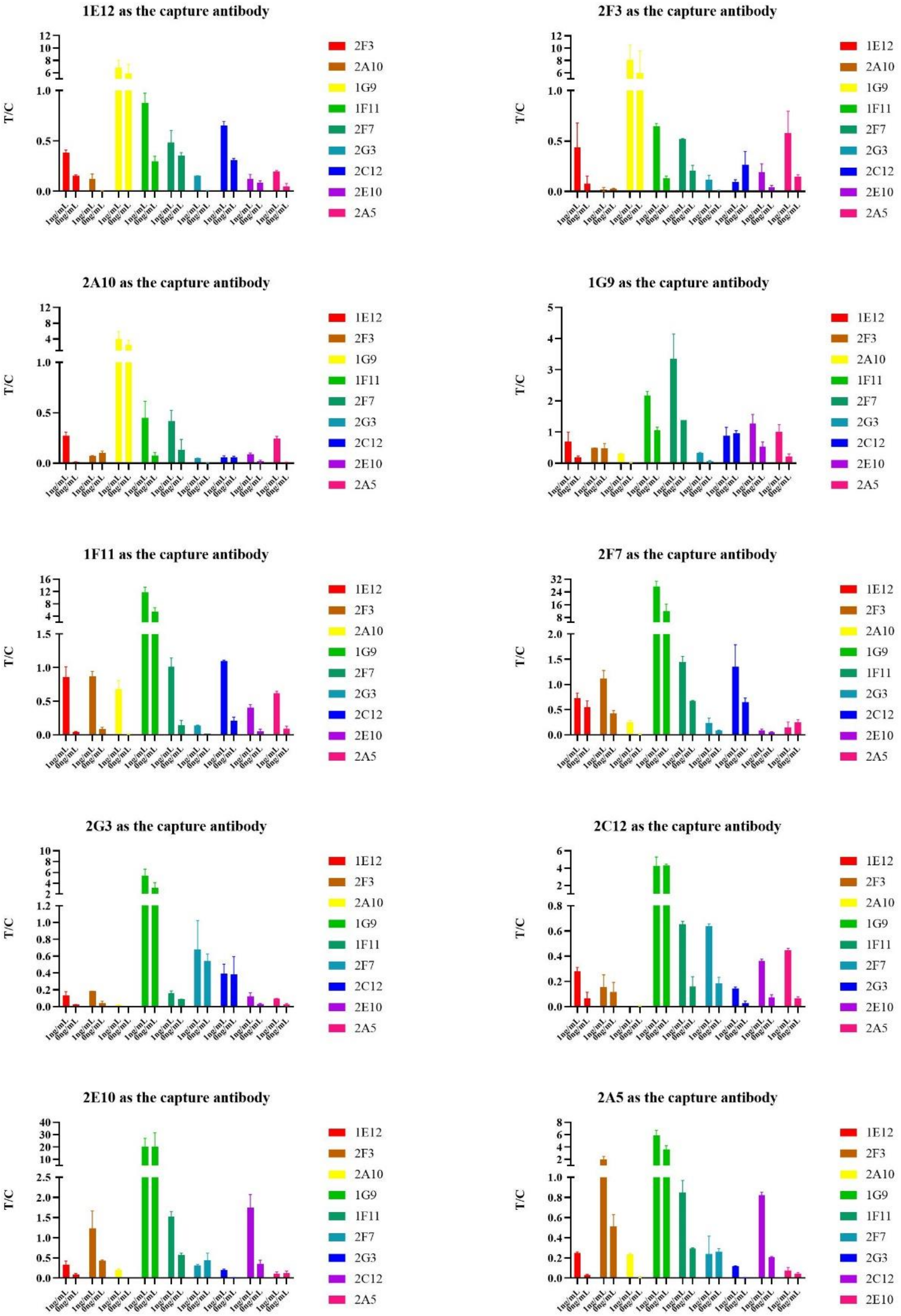

Figure S3 Screening of paired antibodies using TRFIA.
